# Productivity, photosynthetic light-use efficiency, nitrogen metabolism and nutritional quality of C_4_ halophyte *Portulaca oleracea* L. grown indoors under different light intensities and durations

**DOI:** 10.3389/fpls.2023.1106394

**Published:** 2023-02-15

**Authors:** Jie He, Jia Hui Shirin Gan, Lin Qin

**Affiliations:** National Institute of Education, Nanyang Technological University, Singapore, Singapore

**Keywords:** C4 halophyte, light intensity and duration, photosynthetic light-use efficiency, nitrogen metabolism, nutritional quality, productivity, root morphology

## Abstract

*Portulaca oleracea* L. (known as purslane), is a nutritious facultative C_4_ halophyte. Recently, it has been successfully grown indoors under LED lightings by our team. However, basic understanding about the impacts of light on purslanes are lacking. This study aimed to investigate the effects of light intensity and duration on productivity, photosynthetic light use efficiency, nitrogen metabolism and nutritional quality of indoor grown purslanes. All plants were grown in 10% artificial seawater hydroponically under different photosynthetic photon flux densities (PPFDs) and durations and thus different daily light integrals (DLI). They are, L1 (240 µmol photon m^-2^ s^-1^, 12 h, DLI = 10.368 mol m^-2^ day^-1^); L2 (320 µmol photon m^-2^ s^-1^, 18 h, DLI = 20.736 mol m^-2^ day^-1^); L3 (240 µmol photon m^-2^ s^-1^, 24 h, DLI = 20.736 mol m^-2^ day^-1^); L4 (480 µmol photon m^-2^ s^-1^, 12 h, DLI = 20.736 mol m^-2^ day^-1^), respectively. Compared to L1, higher DLI promoted root and shoot growth and thus increased shoot productivity by 2.63-,1.96-, 3.83-folds, respectively for purslane grown under L2, L3, L4. However, under the same DLI, L3 plants (continuous light, CL) had significantly lower shoot and root productivities compared those with higher PPFDs but shorter durations (L2 and L4). While all plants had similar total chlorophyll and carotenoid concentrations, CL (L3) plants had significantly lower light use efficiency (F_v_/F_m_ ratio), electron transport rate, effective quantum yield of PSII, photochemical- and non-photochemical quenching. Compared to L1, higher DLI with higher PPFDs (L2 and L4) increased leaf maximum nitrate reductase activity while longer durations increased leaf ${\text{NO}}_{3}^{-}$ concentrations and total reduced nitrogen. There were no significant differences in leaf total soluble protein, total soluble sugar and total ascorbic acid concentrations in both leaf and stem regardless of light conditions. However, L2 plants had the highest leaf proline concentration but leaf total phenolic compounds concentration was higher in L3 plants instead. Generally, L2 plants had the highest dietary minerals such as K, Ca, Mg and Fe among the four different light conditions. Overall, L2 condition is the most suitable lighting strategy in enhancing productivity and nutritional quality of purslane.

## Introduction

To enhance food security in Singapore, it has been proposed to build our capability and capacity to locally produce 30% of our nutritional needs by 2030 (Singapore Food Agency, 2020). Utilisation of seawater to grow nutritious halophyte vegetables could be a strategy to solve the problem of water shortage in Singapore as halophytes do not compete fresh water with glycophytes. Furthermore, due to the limited available land, we are also developing vertical growth systems to produce vegetables under LED lightings (He et al., 2020b; He et al., 2021; He et al., 2022). *Portulaca oleracea* L. (known as purslane) is one of the most nutritious halophytes owing to its high content of antioxidants (Lim and Quah, 2007; Uddin et al., 2012; Alam et al., 2014). Purslane is a C_4_ halophyte, which performs CAM under drought (Ferrari et al., 2020) and salt stress (He et al., 2021). Recently, we have successfully grown purslane indoors using vertical farming systems with different saline nutrient solutions. In our study, all purslane plants were grown under a photosynthetic photo flux density (PPFD) of 200 μmol m^-2^ s^-1^ (12 h), supplied by red-LED to blue-LED ratio of 2.2 (defined as LED R/B 2.2). Our findings suggest that a 100 mM of NaCl is the most suitable saline condition to grow purslane in order to achieve higher productivity and better quality using our indoor farming systems (He et al., 2021).

For vertical farming, most studies are using different combinations of LED light spectra at low PPFD between 150 to 300 µmol m^-2^ s^-1^ (He et al., 2018; Palmer and van Iersel, 2020). It has been reported that photosynthetic performance, shoot and root biomass accumulation decreased in low light condition (Yang et al., 2018; He and Qin, 2022). For instance, Yang et al. (2018) concluded that light intensity directly affects the expression of photosynthetic proteins which regulate the synthesis of photosynthetic pigments, photosynthetic electron transport and Calvin cycle in soybean (*Glycine max* L. Merr.). Using three different vegetables namely Kai Lan (*Brassica alboglabra*), Nai Bai (*B. chinensis* L.) and mizuna (*B. juncea* var. *japonica)*, we (He and Qin, 2022) carried out comparative studies under different LED spectral qualities and quantities. All plants had much higher shoot fresh weight (FW) under a PPFD of 500 µmol m^-2^ s^-1^ than under a PPFD of 300 µmol m^-2^ s^-1^ regardless of LED combinations. These results suggest that not only LED quality but also intensity is important to enhance shoot productivity (Nájera and Urrestarazu, 2019).

Both light intensity and duration (photoperiod) determine daily light integral, DLI (mol m^-2^ day^-1^), which describes the number of photosynthetically active photons that are delivered to a specific area over a 24-hour period. Many plant growth traits are better related to DLI than to instantaneous PPFD levels at any given time (Poorter et al., 2019). Under artificial light conditions, the light intensity and photoperiod can be varied to determine the optimal DLI used to improve not only crop productivity but also nutritional quality. For instance, sweet basil (*Ocimum basilicum*) grown under higher DLIs of 12.9, 16.5, or 17.8 mol m^-2^ day^-1^ had higher shoot FW, photosynthesis, stomatal conductance and transpiration than under lower DLI of 9.3 mol m^-2^ day^-1^. It was also found increases in soluble sugar and phenolic contents in basil plants grown under higher DLIs than under lower DLIs (Dou et al., 2018). In the study of hydroponically grown lettuce (*Lactuca sativa* L. cv. Ziwei), there was a positive linear correlation between DLI and leaf biomass with increasing tDLI from 6.48 to 17.28 mol m^-2^ day^-1^ (Zhang et al., 2018). These results suggested that a low PPFD could be compensated for a longer photoperiod at the same DLI which could be achieved either by a long duration with a low P PFD or by a short duration with a high PPFD. However, in another study with two different lettuce cultivars, Kelly et al. (2020) reported that specific PPFD and photoperiod combinations can have different effects on plant growth as well as the nutritional quality.

Continuous lighting (CL) maximizes the light duration while minimizes the light intensity at the same DLI. The application of CL in a vertical indoor farm is considered an effective method to save both lighting and cooling costs (Ohyama et al., 2005). However, the effects of CL on plants could be either positive such as increased productivity and enhanced quality (Zha et al., 2019; Elkins and van Iersel, 2020; Proietti et al., 2021) or negative such as induced leaf chlorosis and accelerated senescence (Demers and Gosselin, 2000; Sysoeva et al., 2010; Velez-Ramirez et al., 2011; Shimomura et al., 2020). Compared with 16 h, Zha et al. (2019) found that a greater shoot biomass and antioxidant such as reduced ascorbate and dehydroascorbate contents without leaf injury in hydroponic lettuce were obtained under CL at the same PPFD of 200 µmol m^-2^ s^-1^ provided by red and blue LEDs. In another study with lettuce, Elkins and van Iersel (2020) reported that there are correlations among PPFD, photoperiod, DLI and photosynthetic light use efficiency measured by electron transport through photosystem II (PS II). They found that the total electron transport through PS II would increase if the same DLI was provided at a lower PPFD over 24 hours. In the study with rocket plants (*Eruca vesicaria* L.), CL enhanced yield and nutritional quality measured by pigments, antioxidant compounds. However, nitrate (NO_3_^-^) was significantly reduced in rocket leaves grown under CL. This result implies that CL may increase nitrate reductase activity (NRA) and the reduction of ${\text{NO}}_{3}^{-}$ (Proietti et al., 2021). In the study of medicinal plant, Nasturtium (*Tropaeolum majus* L.) under the same DLI but different photoperiods with different PPFDs, Xu et al. (2021) found that leaf biomass, concentrations of secondary metabolites, and light use efficiency increased under the CL compared to the 16-h treatment. However, Demers and Gosselin (2000) reported that yield and chlorophyll (Chl) concentration decreased when tomato and sweet pepper plants were subjected to CL. The negative effects of CL on certain species may be due to the photooxidative damage caused by CL. Shimomura et al. (2020) reported that the highest antioxidant content such as chlorogenic acid (CGA) of lettuce was obtained under the CL treatment, suggesting that CGA protected plants from photooxidative damage caused by CL.

Compositions and concentrations of bioactive compounds of purslane can be affected by both light intensity and photoperiod (Palaniswamy et al., 2001; Petropoulos et al., 2016). Given that purslane is a C_4_ plant, it is able to adapt to high light conditions *via* the C_4_ photosynthetic pathway (Gonnella et al., 2010). Under high light intensities, C_4_ plants have higher photosynthetic rates and higher productivity than C_3_ plants (Pearcy and Ehleringer, 1984). It was reported that purslane can thrive in various light intensities as well as different photoperiods (Singh, 1972; Koch and Kennedy, 1980; Ferrari et al., 2020). However, a very little research has been done on the effects of light intensity, duration and DLI on growth, physiological performance and nutritional quality of purslane grown indoors. Our hypothesis is that high light intensity and higher DLI could enhance productivity and improve nutritional quality of C_4_ purslane grown indoors. We also tested if the same DLI was provided at a lower PPFD through CL could increase productivity and enhance nutritional quality compare to those plants grown with the same DLI under higher PPFDs but shorter durations. The findings of the study could provide lighting strategy for purslane growers to enhance its productivity and nutritional quality through the selection of light intensity and duration.

## Materials and methods

### Plant materials and experimental design

Seeds of purslane (*P. oleracea* L. cv. POR – 2936) were used in this study. Seedlings were inserted into polyurethane cubes after germination for 4 days. All seedlings were placed under a PPFD of 100 μmol m^−2^ s^−1^ supplied by high-pressure sodium lamps for 4 weeks before transplanting onto indoor hydroponic systems. Plants were then grown in 10% artificial seawater (ASW) with full strength nutrient solution. To make up a 10% ASW, a salinity of 3.3 ppt was prepared by dissolving 28.8g of Red Sea Salt^®^ (Red Sea Fish Pharm Ltd., Eilat, Israel; www.redseafish.com) in 8 L full strength nutrient solution. The conductivity of and the pH of the nutrient solutions were 2.2 ± 0.2 mS cm^−1^ and 6 ± 0.2, respectively. The plants were grown under four different light intensities and durations: L1 (240 µmol photon m^-2^ s^-1^, 12 h, DLI = 10.368 mol m^-2^ day^-1^); L2 (320 µmol photon m^-2^ s^-1^, 18 h, DLI = 20.736 mol m^-2^ day^-1^); L3 (240 µmol photon m^-2^ s^-1^, 24 h, DLI = 20.736 mol m^-2^ day^-1^); L4 (480 µmol photon m^-2^ s^-1^, 12 h, DLI = 20.736 mol m^-2^ day^-1^). The same LED spectral quality with red/blue LED ratios of 2.2 (WR-16W, Beijing Lighting Valley Technology Co., Ltd., China) was used for all the different light conditions. The light spectral distribution was reported by He et al. (2021). The room temperature and relative humidity were 25*°*C/23*°*C and 60%/80% (day/night) respectively.

### Root morphology

After removing the polyurethane cubes, the roots were spread out in a tray of water. The roots were first scanned using the WIN MAC RHIZO scanner. Subsequently, the WIN MAC RHIZO V 3.9 programme was used to analyse the root morphological parameters including total root length, number of root tips, total root surface area and average root diameter.

### Productivity, leaf growth and leaf water status

On day 8 and 16 after transplanting, the whole plants were harvested. After removing the polyurethane cubes from the roots, shoot (leaf and stem) and root were separated for FW measurements. The total leaf number was recorded before determining the total leaf area (TLA) of each plant using leaf area meter (WinDIAS3 Image Analysis system). To obtain DW, shoot and roots were wrapped separately in aluminium foils and dried in the oven at 80°C for 4 days. Specific leaf area (SLA) was calculated using leaf area (cm^2^)/leaf dry weight (g) (Hunt et al., 2002).

### Chl fluorescence F_v_/F_m_ ratio, electron transport rate (ETR), effective quantum yield of PSII (Δ*F/F*_m_′), photochemical quenching (qP) and non-photochemical quenching (NPQ)

F_v_/F_m_ ratios (maximum potential quantum efficiency of PSII) of dark-adapted attached leaves were measured using the Plant Efficiency Analyser (Hansatech Instruments Ltd, England) during mid-photoperiod. The ETR, ΔF/F_m_’, qP and NPQ were determined from detached leaf using the IMAGING PAM MAXI (Walz, Effeltrich, Germany) at 25°C in the laboratory. The details of F_v_/F_m_ ratio, ETR, Δ*F/F*_m_′, qP and NPQ measurements were described by He et al. (2011).

### Measurements of total Chl and carotenoids (Car) concentrations

Leaf samples (0.05 g) were harvested on day 12 after transplanting and soaked in 5 ml of N, N-dimethylformamide in the dark for 48 h at 4°C (Wellburn, 1994). The absorbance was read at wavelength of 480, 647 and 664 nm, respectively. The concentrations of *Chl a*, *b*, and Car were calculated according to Wellburn (1994).

### Measurements of NO_3_^-^ and total reduced nitrogen (TRN) concentration

Plant samples were dried at 80°C for 4 days. Dried samples (0.01 g) were grounded with 10 ml deionised water and were then incubated at 37°C for two hours. Sample turbidity was then removed by vacuum filtering the mixture through a 0.45 µm-pore-diameter membrane. The flow injection analyser (Model Quikchem 800, Lachat Instruments Inc., Milwaukee, USA) was used to measures ${\text{NO}}_{3}^{-}$ concentration through the reduction of NO_3_^-^ to nitrite ( ${\text{NO}}_{2}^{-}$ ) when the sample passes through a copperized cadmium column. ${\text{NO}}_{2}^{-}$ was diazotized with sulphanilamide and coupled with N-(1-naphthyl) ethylenediamine dihydrochloride resulting in a magenta water-soluble dye that was read at 520 nm. TRN content was determined by Kjeldahl digestion of 0.05 g of dried samples and a Kjeldahl tablet in 5 ml of concentrated sulphuric acid for 60 min at 350°C. After digestion, TRN concentration was quantified by Kjeltec 8400 analyzer (Foss Tecator AB, Höganäs, Sweden) through titration.

### Measurements of maximum NR activity (NRA_max_) and NR activation state (NR_act_)

The frozen sample stored at −80°C was powdered in liquid nitrogen and ground with 4 ml of ice-cold extractiPon buffer with the presence of 0.2 g/g FW PVPP. The extraction buffer included 0.25 M Tris-HCl (pH 8.5), 3 mM dithiothreitol (DTT), 10 µM flavin adenine dinucleotide (FAD), 1 µM sodium molybdate, 1 mM Ethylenediamine-tetra-aceticacid (EDTA). The extracts were centrifuged at 18,000 g for 20 min at 4°C. The supernatant was used to determine the NRA immediately.

*In vitro* NADH:NRA assay was derived from Kaiser and Huber (1997) with modification. The NR_act_ was determined by assaying NR either with Mg^2+^ (10 mM), or with EDTA (15 mM). In all cases, the total reaction medium was 700 µl which contained 50 mM Hepes-KOH (pH 7.5), 1 mM DTT, 10 µM FAD, 10 mM KNO_3_, 0.2 mM NADH, NR extraction, and 10 mM MgCl_2_ or 15 mM EDTA. The reaction was started by adding of 100 – 200 µl NR extraction. Incubation was performed at 25°C for 20 min, and the reaction was then terminated by the addition of an equal volume (700µl) of Sulfanilamide (1%(w/v) in 3 N HCl) and the naphthylethylens-diamine dihydrochloride (0.02% w/v). After 30 min at room temperature, the absorbance was read at wavelength of 540nm. The blank was identical to the samples, but the NR extracts were boiled for 5 min before the addition. NRA_max_ was expressed as µmol nitrite ( ${\text{NO}}_{2}^{-}$ ) h^-1^ g^-1^ FW. The NR_act_ is defined as the activity measured in the presence of 10 mM MgCl_2_ divided by the activity measured in the presence of 15 mM EDTA (expressed as a percentage).

### Measurements of leaf total soluble protein (TSP) concentration

The details of leaf TSP extraction were described in He et al. (2017a). The frozen samples (1 g) were powdered in liquid nitrogen and ground with 6 ml of extraction buffer [100 mM Bicine-KOH (pH 8.1), 20 mM MgCl2, 2% PVP]. The mixture was centrifuged at 35,000 rpm for 30 min at 4°C using a Beckman ultracentrifuge Optima XL-100K. 1 ml aliquot of the supernatant was mixed with 4 ml of 80% cold acetone before centrifuging at 3500 rpm for 10 min. The precipitate was dissolved in 1 ml of 1 M NaOH. The concentration of leaf TSP was determined according to Lowry et al. (1951).

### Determination of total soluble sugars (TSS)

Dried plant tissues of 0.01 g were added to 4 ml of 80% ethanol and heated in a 65°C water bath for 30 min. The homogenate was centrifuged at 4000 rpm for 5 min before collecting the supernatant. The pellet was resuspended with another 2 ml of 80% ethanol and the process was repeated twice. TSS concentration was determined at 490 nm using a spectrophotometer (UV-2550 Shimadzu, Japan) according to Dubois et al. (1956).

### Determinations of proline, ascorbic acids (ASC), and total phenolic compounds (TPC)

The same amount of froze plant tissues (0.5 g) was used to extract proline, ASC and TPC, separately. The details of extraction processes for these three phytochemicals were described in He et al. (2020b). The proline assay was modified from Bates et al. (1973). The absorbance of proline extract was measured at 520 nm. For ASC and TPC, the absorbances were determined at 524nm and 765 nm, respectively based on Leipner et al. (1997) and Ragaee et al. (2006). All absorbances were measured using a spectrophotometer (UV-2550 Shimadzu, Japan).

### Determination of dietary minerals

Dried samples (0.2 g) were digested in 4 ml of 65% nitric acid using UltraWAVE single reaction chamber microwave digestion system (Milestone, US). The digested solution was diluted with Milli-Q water to a final volume of 25 ml. The Optima 8300 ICP-OES (Inductively Coupled Plasma Optical Emission Spectrophotometer) and Syngistix software (Perkin Elmer, US) were used to measure and calculate the concentrations of dietary minerals.

### Statistical analysis

One-way (ANOVA) was used to test for significant differences among the different treatments. Tukey’s multiple comparison tests were carried out to discriminate among the means of the different groups (MINITAB 19).

## Results

### Root morphology

**Figure 1:**
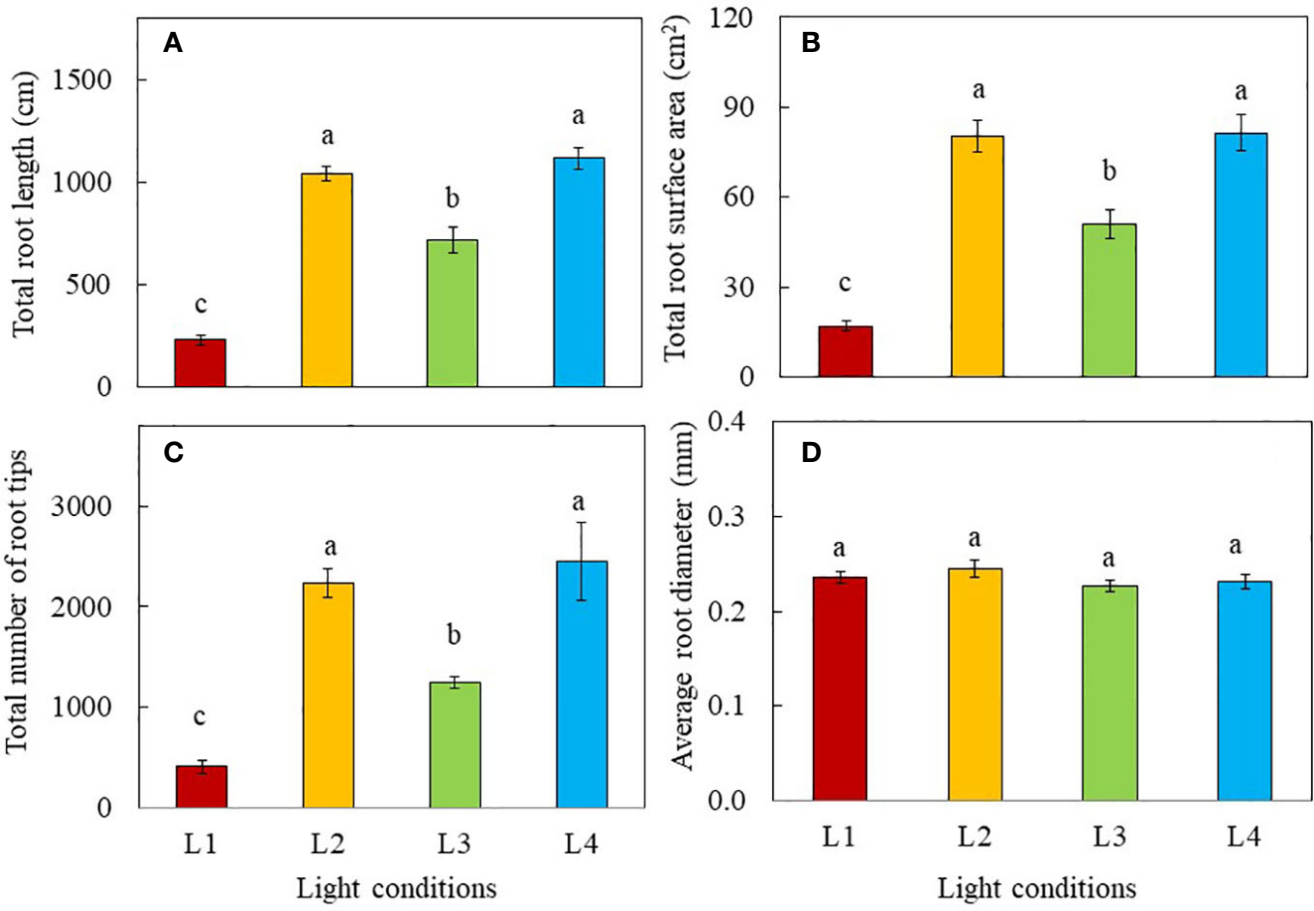
Total root length **(A)**, total root surface area **(B)**, total number of root tips **(C)** and average root diameter **(D)** of purslane grown under different light treatments for 8 days. Values are means ± standard errors. Means with different letters are statistically different (*p*<0.05; n =5) as determined by Tukey’s multiple comparison test. L1 (240 µmol photon m^-2^ s^-1^, 12 h, DLI = 10.368 mol m^-2^ day^-1^); L2 (320 µmol photon m^-2^ s^-1^, 18 h, DLI = 20.736 mol m^-2^ day^-1^); L3 (240 µmol photon m^-2^ s^-1^, 24 h, DLI = 20.736 mol m^-2^ day^-1^); L4 (480 µmol photon m^-2^ s^-1^, 12 h, DLI = 20.736 mol m^-2^ day^-1^).

Root morphology was analysed 8 days after transplanting and the results are shown in **Figure 1**. The total root length, total root surface area and total number of root tips of L2 (320 µmol photon m^-2^ s^-1^, 18 h, DLI of 20.736 mol m^-2^ day^-1^) and L4 (480 µmol photon m^-2^ s^-1^, 12 h, DLI of 20.736 mol m^-2^ day^-1^) plants were similar but significantly greater than those of L1 (240 µmol photon m^-2^ s^-1^, 12 h, DLI of 10.368 mol m^-2^ day^-1^) and L3 plants (240 µmol photon m^-2^ s^-1^, 24 h, DLI of 20.736 mol m^-2^ day^-1^). (**Figures 1A–C**). There were no significant differences in average root diameter among light treatments (**Figure 1D**).

### Productivity and leaf growth traits

**Figure 2:**
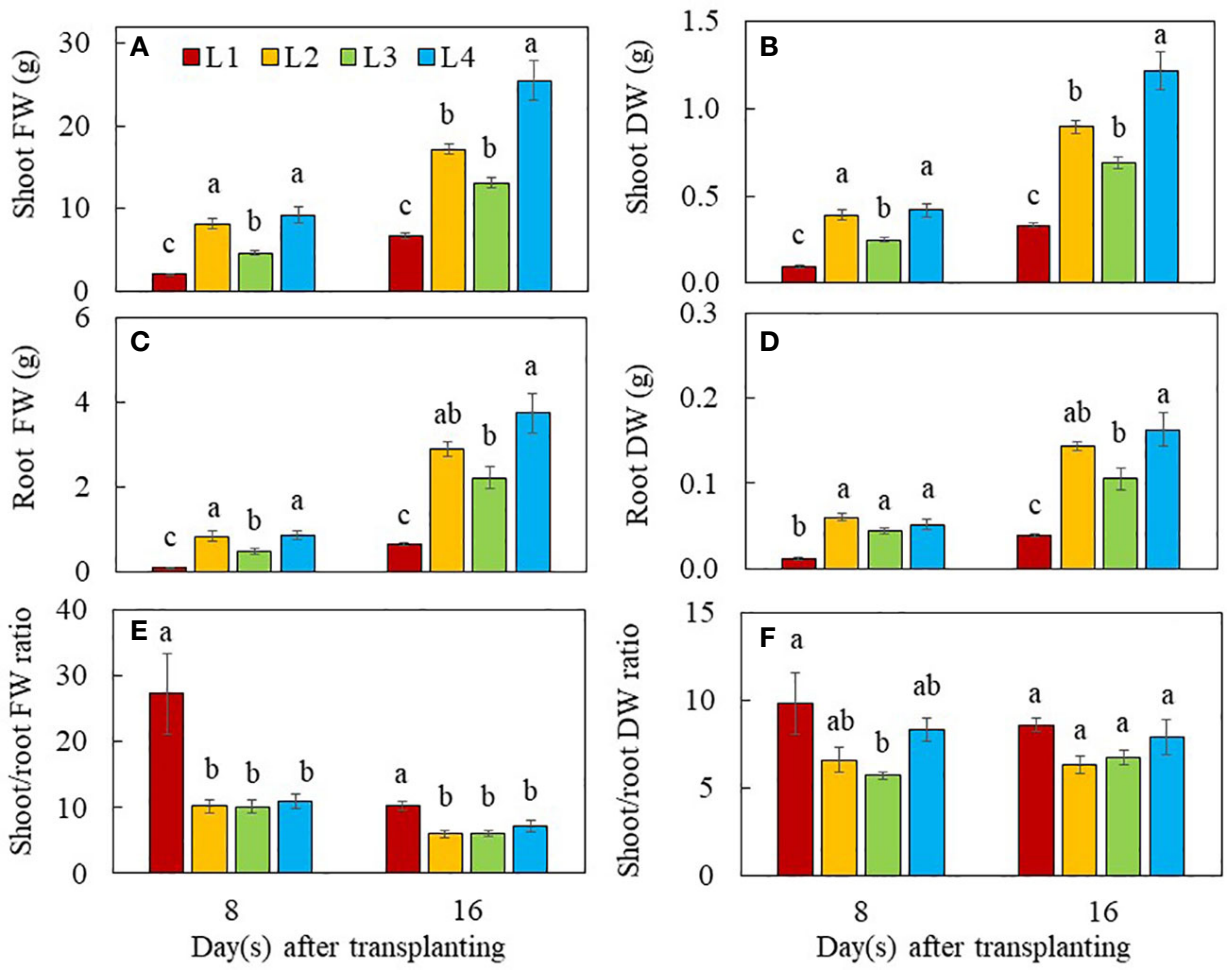
Shoot FW **(A)**, shoot DW **(B)**, root FW **(C)**, root DW **(D)**, shoot/root FW ratio **(E)** and shoot/root DW ratio **(F)** of purslane grown under different light conditions for 8 and 16 days. Values are means ± standard errors. Means with different letters are statistically different (*p*<0.05; n =5) as determined by Tukey’s multiple comparison test for each day. Refer to **Figure 1** for the different conditions of L1, L2, L3 and L4.

**Figure 3:**
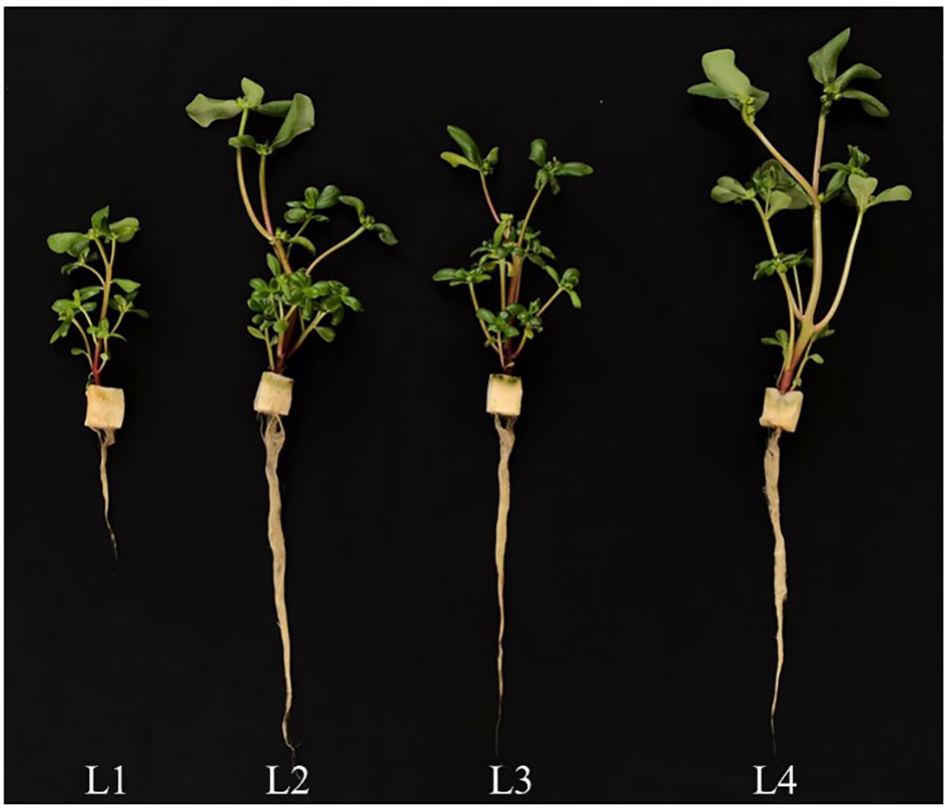
Purslane plants grown indoors hydroponically under LED lighting (R/B ratio of 2.2) with different light intensities and durations for 16 days. Refer to **Figure 1** for the different conditions of L1, L2, L3 and L4.

On day 8 after transplanting, the shoot FW (**Figure 2A**) and DW (**Figure 2B**) as well as root FW (**Figure 2C**) were significantly higher in L2 and L4 plants than L1 and L3 plants. For root DW, L2, L3 and L4 plants were similar but significantly higher than that of L1 plants (**Figure 2D**). At the early stage of 8 days after transplanting, L1 plants grown under the lowest PPFD with the shortest photoperiod with the lower DLI of 10.368 mol m^-2^ day^-1^ had the lowest shoot and root productivity compared to those of plants grown under higher PPFD with same duration (L4) or higher PPFD with longer duration (L2 and L3 but with the same DLI of 20.736 mol m^-2^ day^-1^). Grown under the same DLI, L2, and L4 plants with non-CL had significantly higher shoot and root productivity than L3 plants with CL. On day 16 after transplanting, however, L4 plants had the highest values for shoot and root productivity, followed by L2 and L3 plants. The L1 plants had the lowest values but no significant differences were observed between L2 and L3 plants (**Figures 2A–D**). The lowest shoot FW and DW of L1 plants were mainly due to the lowest branch number, stem height and total number of leaves as well as the smallest total leaf area compared to those of L2, L3 and L4 plants (**Figure 3**). It was noted that the shoot/root FW ratio of L1 plants was the highest on day 8 and 16 after transplanting, almost twice the value than the other groups (**Figure 2E**). On day 8 after transplanting, the shoot/root DW ratio was the highest in L1 plants and the lowest in L3 plants, while shoot/root DW ratios of L2 and L4 plants did not significantly differ. On day 16 after transplant, no significant differences in shoot/root DW ratios were found among light treatments (**Figure 2F**).

**Figure 4:**
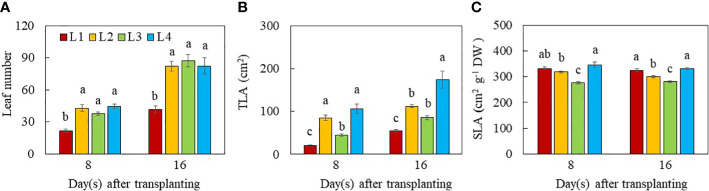
Leaf number **(A)**, TLA **(B)** and SLA **(C)** of purslane grown under different light conditions for 8 and 16 days. Values are means ± standard errors. Means with different letters are statistically different (*p*<0.05; n =5) as determined by Tukey’s multiple comparison test for each day. Refer to **Figure 1** for the different conditions of L1, L2, L3 and L4.

The leaf number of L1 plants was significantly lower than all other groups on both day 8 and 16 after transplant (**Figure 4A**), indicating that plants with higher DLI increased leaf number regardless of light intensity and duration. On day 8 after transplanting, the total leaf area (TLA) was significantly higher in L2, L3 and L4 plants than in L1 plants. On day 16 after transplanting, however, L4 had the highest TLA, followed by L2 and L3, with L1 plants having the lowest values. The TLA of L3 plants did not significantly differ from L2 plants on day 16 after transplanting (**Figure 4B**). The SLA was the highest in L1 and L4 plants, followed by L2, with L3 plants having the lowest values on day 8 and 16 after transplanting. On day 8 after transplant, the SLA of L1 and L2 plants did not significantly differ (**Figure 4C**).

### Photosynthetic pigments, F_v_/F_m_ ratio, ETR, ΔF/F_m_’, qP and NPQ

**Figure 5:**
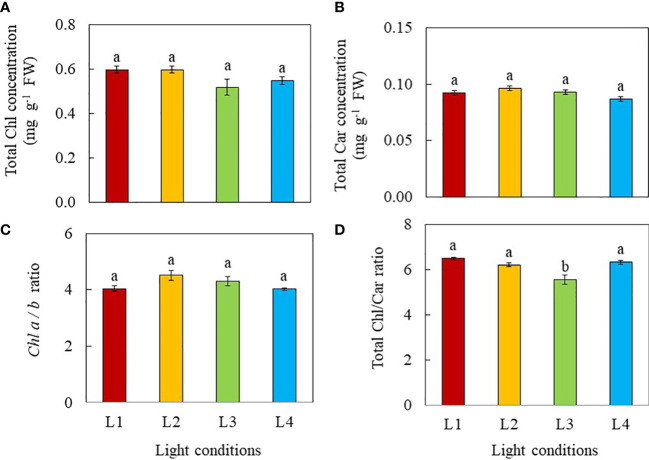
Total Chl concentration **(A)**, total Car concentration **(B)**, *Chl a/b* ratio **(C)** and total Chl/Car ratio **(D)** of purslane grown under different light conditions for 12 days. Values are means ± standard errors. Means with different letters are statistically different (*p*<0.05; n =5) as determined by Tukey’s multiple comparison test. Refer to **Figure 1** for the different conditions of L1, L2, L3 and L4.

The total Chl, total Car concentrations and the *Chl a/b* ratio were not significantly different among the different light treatments (**Figures 5A–C**). However, the Chl/Car ratio of L3 plants grown under CL condition was significantly lower than that of all other three light treatments (**Figure 5D**).

**Figure 6:**
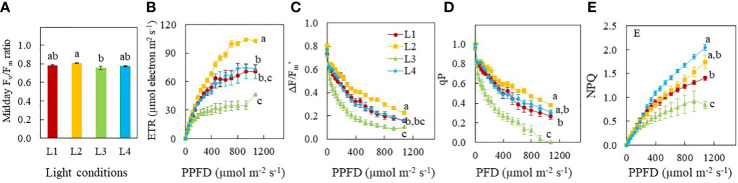
F_v_/F_m_ ratio **(A)** of Purslane (n=8) grown under different light treatments for 9 days. Light response curves of ETR **(B)**, ΔF/F_m_’ **(C)**, qP **(D)** and NPQ **(E)** of purslane (n=5) grown under different light conditions for 12 days. Values are means ± standard errors. Means with different letters are statistically different (*p*<0.05) as determined by Tukey’s multiple comparison test. Refer to **Figure 1** for the different conditions of L1, L2, L3 and L4.

During mid-photoperiod, the F_v_/F_m_ ratios of L1, L2 and L4 plants were around or close to 0.8 and the F_v_/F_m_ ratios of L2 plants was significantly higher than the F_v_/F_m_ ratio of L3 plants. The F_v_/F_m_ ratio of 0.754 for L3 plants, indicates mild light stress resulting from CL (**Figure 6A**). Measured at a PPFD of 1076 m^-2^ s^-1^, ETR, ΔF/F_m_’ and qP were the highest in L2 plants, followed by L1 and L4 plants and the lowest values were found in L3 plants (**Figures 6B–D**). However, at a PPFD of 1076 m^-2^ s^-1^, NPQ values were the highest in plants grown under L4, followed by L2 and L1 with L3 having the lowest value (**Figure 6E**).

### N metabolism

**Figure 7:**
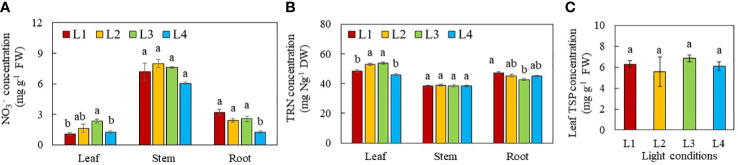
NO_3_^-^ **(A)**, TRN **(B)** and TSP **(C)** concentrations of purslane grown under different light conditions for 13 days. Values are means ± standard errors. Means with different letters are statistically different (*p*<0.05; n =4) as determined by Tukey’s multiple comparison test for each plant organ. Refer to **Figure 1** for the different conditions of L1, L2, L3 and L4.

**Figure 8:**
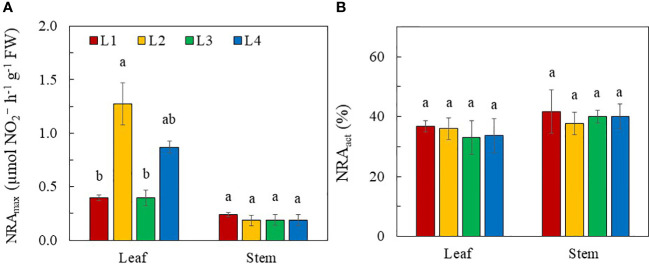
NRA_max_ **(A)** and NR_act_ **(B)** of purslane grown under different light conditions for 14 days. Values are means ± standard errors. Means with different letters are statistically different (*p*<0.05; n =3) as determined by Tukey’s multiple comparison test for each plant organ. Refer to **Figure 1** for the different conditions of L1, L2, L3 and L4.

The effects of light conditions on N metabolism were studied by the measurements of ${\text{NO}}_{3}^{-}$ (**Figure 7A**) and TRN concentrations in leaf, stem and root (**Figure 7B**). as well as NRA in leaf and stem (**Figure 8**). The leaf TSP was also determined (**Figure 7C**). For all light treatments, NO_3_^-^ concentration was lower in the leaves than the stem and roots. Leaf NO_3_^-^ concentration was the highest in L3 plants and the lowest in L1 and L4 plants. Leaf NO_3_^-^ concentration in plants grown under L2 did not significantly differ from the other groups. There were no significant differences in stem NO_3_^-^ concentration among the plants grown under different light treatments. The root NO_3_^-^ concentration of plants grown under L4 was significantly lower than that of all other light treatments (**Figure 7A**). All plants had similar TRN concentration in their stems. For leaves, the TRN concentrations were significantly higher in L2 and L3 leaves than those of L1 and L4 leaves. For the roots, L1 plants had significantly higher TRN than L3 plants which TRN concentration was not significantly different from those of L2 and L4 plants (**Figure 7B**). There were no significant differences in leaf TSP concentration among purslane grown under different light conditions (**Figure 7C**).

All plants had higher NRA_max_ in the leaves compared to the stems. NRA_max_ was hardly detected in the roots. Leaf NRA_max_ was much higher in L2 and L4 plants than those in L1 and L3 plants although statistically there was no significant difference in leaf NRA_max_ between L3 and L4 plants. All plants had a similar stem NRA regardless of light conditions (**Figure 8A**). The NR_act_ was not correlated with NRA_max_ in leaves as there were no significant differences in NR_act_ in both leaves and stems among plants grown under different light treatments (**Figure 8B**).

### Nutritional quality

**Figure 9:**
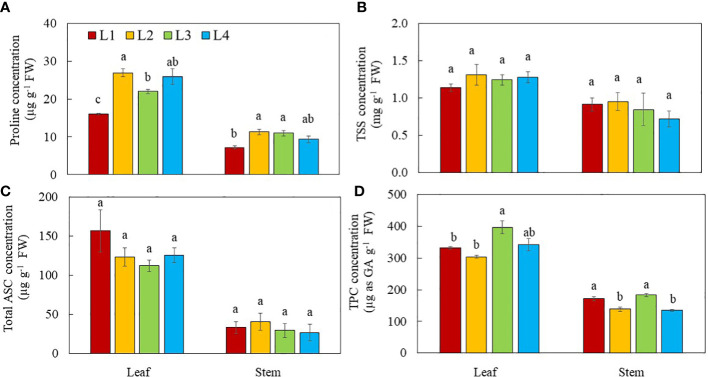
Proline **(A)**, TSS **(B)**, total ASC **(C)** and TPC **(D)** of purslane grown under different light conditions for 14 days. Values are means ± standard errors. Means with different letters are statistically different (*p*<0.05; n =4) as determined by Tukey’s multiple comparison test for each plant organ. Refer to **Figure 1** for the different conditions of L1, L2, L3 and L4.

Proline, TSS, total ASC and TPC concentrations were higher in the leaves than the stems regardless of light conditions (**Figure 9**). Leaf proline concentrations were higher in L2 and L4 plants than in L3 plants. L1 plants had the lowest leaf proline concentration. Stem proline concentration in L1 plants was the lowest compared to other groups (**Figure 9A**). For both leaf and stem, no significant differences in TSS (**Figure 9B**) and total ASC (**Figure 9C**) concentrations were observed among the plants grown under different light conditions. Leaf TPC concentration was higher in L3 plants than other groups, while leaf TPC in L4 plants did not significantly differ from other groups. For stems, the TPC concentration was higher in L3 and L1 plants than those of L2 and L4 plants (**Figure 9D**).

**Figure 10:**
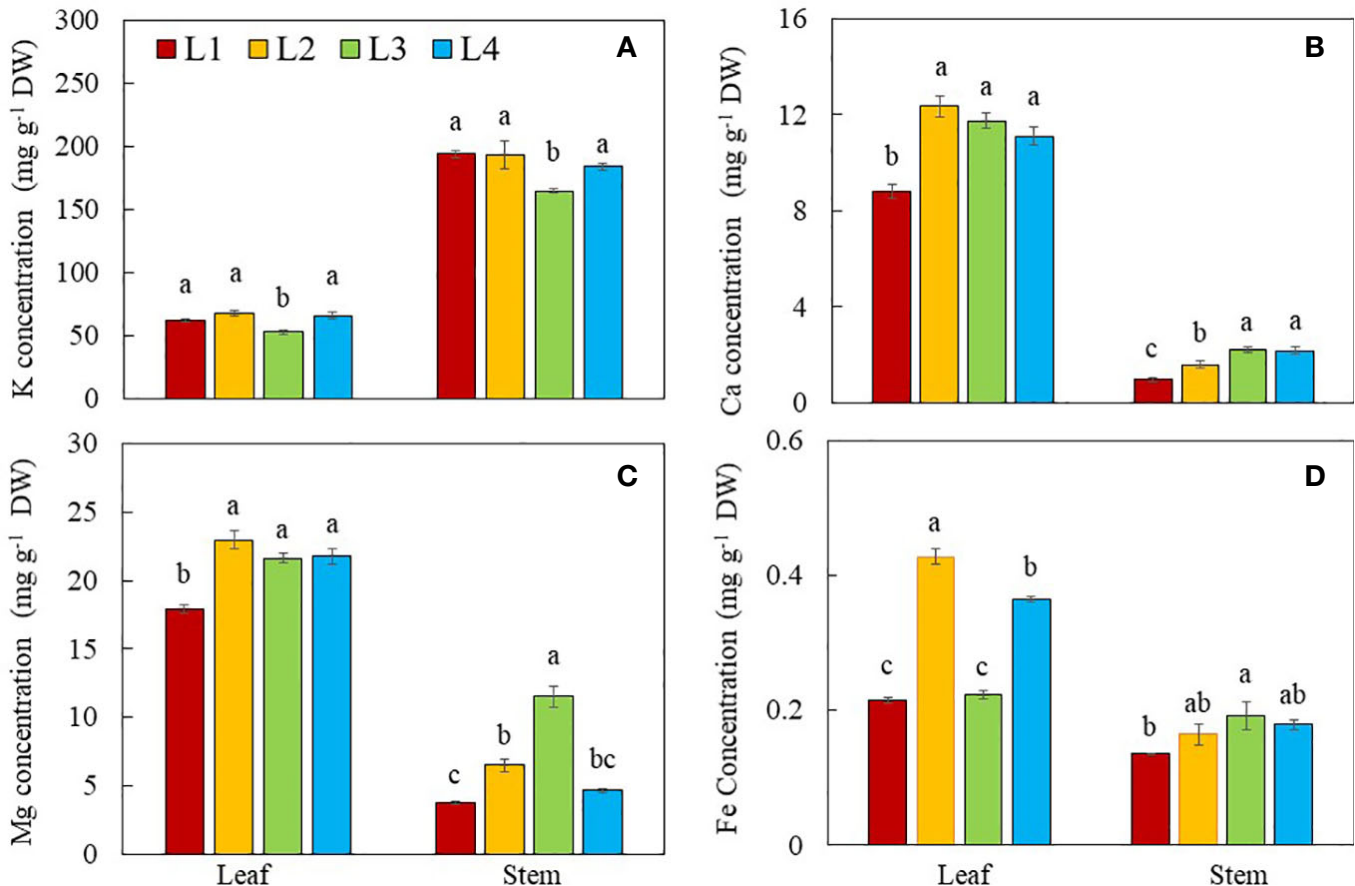
Dietary minerals, K **(A)**, Ca **(B)**, Mg **(C)** and Fe **(D)** concentrations of purslane grown under different light conditions for 14 days. Values are means ± standard errors. Means with different letters are statistically different (*p*<0.05; n =4) as determined by Tukey’s multiple comparison test for each plant organ. Refer to **Figure 1** for the different conditions of L1, L2, L3 and L4.

With the exception of K, a higher concentration of Ca, Mg and Fe were found in the leaves compared to those of stems for all plants (**Figure 10**). Stem contained a higher concentration of K, almost double the values found in the leaves. Both leaf and stem K concentrations were significantly lower in L3 plants compared to other groups (**Figure 10A**). Leaf Ca concentration in L1 plants was significantly lower compared to those of L2, L3 and L4 plants. Stem Ca concentration was the highest in plants grown under L3 and L4, followed by L2 and the lowest value was found in L1 plants (**Figure 10B**). For the leaf Mg, L1 plants had significantly lower concentration compared to other groups. Stem Mg concentration was the highest in plants grown under L3 followed by L2, L4 and L1 conditions (**Figure 10C**). Leaf Fe concentration was the highest in L2 plants, followed by L4 plants, with L1 and L3 plants having the lowest values. Stem Fe concentration was the highest in L3 plants and lowest in L1 plants, while values of L2 and L4 plants did not significantly differ (**Figure 10D**).

## Discussion

### Root morphology, productivity of shoot and root, and leaf growth

The aerial parts of the plant have naturally been the main focus in many studies of interactions of plants with light, given that such interactions happen mostly above-ground. However, light provides not only energy to plants in their production of sugars through photosynthesis, but also provides information such as light signaling *via* sugars (Kircher and Schopfer, 2012; Zheng et al., 2019), and phytohormones for root development (Swarup et al., 2008; Costigan et al., 2011). In the “Update on light signaling and root development”, van Gelderen et al. (2018) concluded that light quality and duration impact root physiology and development. In this study, the total root length, total root surface area and total number of root tips were similarly but significantly higher in L2 and L4 plants grown under higher PPFDs compared to those of L3 and L1plants (**Figures 1A–C**). Root morphology is modulated by signals such as photosynthates, phytohormones and more recently, proteins, transmitted across long-distance from aboveground tissues (Lee et al., 2017; van Gelderen et al., 2018). In the presnt study, purslane grown under higher PPFD and higher DLI had larger root system could be resulted from higher amounts of sugars transported to the roots promoting their development (Kircher and Schopfer, 2012; Zheng et al., 2019). Furthermore, high light induced the synthesis of auxin in young leaves. Auxin is polarly transported to the roots and promotes primary and lateral roots development (Swarup et al., 2008; Costigan et al., 2011). Although L3 plants were exposed to the same amount of DLI (20.736 mol m^-2^ day^-1^) as those of L2 and L4, they had shorter total root length, smaller total root surface area and fewer number of root tips than those of L2 and L4 plants (**Figures 1A–C**). This could be due to CL exposure which may result in a sustained increase in cytosolic hexose, triggering starch accumulation in leaves. This inhibits triosephosphate exporting out from the chloroplast to be utilised for growth and development (Demers and Gosselin, 2000), limiting not only root but also shoot biomass accumulation in L3 plants. This speculation was supported by the lower shoot FW and DW, and root FW (**Figures 2A–C**) as well stem height (**Figure 3**) of L3 plants compared to those of L2 and L4 plants on day 8 after transplant. It also further exemplifies the influence of light levels and CL on the productivity of both shoot and root. On day 16 after transplant, L4 plants under the highest PPFD had the highest values of growth parameters, followed by L2 and L3, with L1 grown under the lowest PPFD and shortest photoperiod and thus lower DLI having the lowest values (**Figures 2A–D**). This result suggests that light intensity is a more significant factor affecting plant productivity at a later stage of development of C_4_ purslane. On day 8 and 16 after transplanting, the shoot/root FW ratio of L1 plants was the highest (**Figure 2E**), a trend which was also observed in the shoot/root DW ratios of L1 plants 8 days after transplant (**Figure 2F**). These results imply that more biomass is allocated to the shoots to increase light acquisition (Garnier, 1991). On day 16 after transplanting, no significant differences in shoot/root DW ratios were found in plants grown under different light conditions. This result implies that the effect of low DLI on shoot/root resource allocation is more significant at the early growth stage in L1 plants which were grown under lower DLI.

Leaf growth and development is also regulated by light intensity and photoperiod and thus DLI (Dou et al., 2018; Yan et al., 2019; Camejo et al., 2020; He and Qin, 2020). It was reported that lower light intensity and shorter photoperiod resulted in lower leaf number (Camejo et al., 2020) and smaller total leaf area (Dou et al., 2018). In this study, L1 plants grown under the lowest PPFD and shorter photoperiod had the lowest leaf number and smallest TLA compared to those L2, L3 and L4 plants at both growth stages (**Figure 4A, B**). These results also suggest that an increase in light radiation for L2, L3 and L4 plants compared to L1 plants by increasing DLI could result in the increases of leaf number and TLA at both growth stages. Growing green butterhead lettuce and red oakleaf lettuce under DLIs from 6.9–15.6 mol m^−2^ d^−1^ under PPFDs of 120 to 270 μmol m^–2^ s^–1^ for durations of 16 to 24 h, Kelly et al. (2020) reported that shoot FW and DW, leaf width and number of leaves increased with increasing DLI for both cultivars, irrespective of PPFD and photoperiod combinations. In the study with plantlets of sweet potato (cv. Beniharuka), leaf number was not significantly affected with increasing PPFD from 150 to 350 μmol m^-2^ s^-1^ (He et al., 2020a). However, the TLA of sweet potato plantlets exhibited an increasing trend, but it decreased when the PPFD was greater than 250 μmol m^-2^ s^-1^. These results suggest that a too low or too high PPFD inhibited leaf expansion. Similar result was also reported by Pennisi et al. (2020) in lettuce and basil. However, no difference in TLA or leaf area per leaf were observed among the different photoperiod treatments in either lettuce or mizuna while both species had greater light interception and the quantum yield of PSII with longer photoperiods (Palmer and van Iersel (2020). These results suggest that leaf morphological traits must be due to the differences in the percentage of light interception. We have previously reported that productivity of lettuce plants under different combinations of LED spectra was associated with leaf traits (He et al., 2019). Pennisi et al. (2020) observed a reduction of SLA of basil grown under increased PPFD associated with thicker and larger leaves, which was also reported by Dou et al. (2018) in basil plants. In this study, L1 and L4 plants grown under the lowest and the highest PPFD, respectively for the same photoperiod (12 h), had similar higher SLA compared to L2 and L3 plants while SLA was the lowest in L3 plants at both growth stages (**Figure 4C**). This result suggests that increased PPFD greater than 240 μmol m^-2^ s^-1^, did not affected the leaf thickness. However, when exposed to longer photoperiod (L2 and L3), plants increase the thickness of leaves by synthesising more palisade cell layers to prevent light damage caused by excessive light energy (Wentworth et al., 2006). Depending on species, the SLA and aboveground biomass could be either negatively or positively correlated (Liu et al., 2017). Although L3 plants had lower SLA (**Figure 4C**) or thicker leaves, their smaller TLA (**Figure 4B**) and shorter stem (**Figure 3**) resulted in lower shoot productivity (**Figure 2A**).

Summing up, the above discussion suggests that light intensity, duration and DLI all play important roles in enhancing plant growth and productivity in both early and later stages of growth (Dou et al., 2018; Zhang et al., 2018; Poorter et al., 2019; Kelly et al., 2020). In the study with lettuce, Kelly et al. (2020) reported that at a higher DLI with lower PPFD and longer photoperiod increased growth more than a higher PPFD and shorter photoperiod. However, in this study, under the same DLI, the C_4_ purslane grown under L4 condition with higher PPFD and shorter photoperiod had higher shoot FW compared to those grown under L2 condition with lower PPFD and longer photoperiod for 16 days. Furthermore, under the same DLI, CL (L3) with lower PPFD had lower productivity compared to L2 and L4 conditions which had higher PPFDs but shorter photoperiods. Controversial results such as CL promoted growth were reported in lettuce (Zha et al., 2019), medicinal plant, Nasturtium (Xu et al., 2021) and rocket plants (Proietti et al. (2021). The effectiveness of CL seems depending on plant species as well as the light intensity, duration and dynamic LED lighting strategies (Pham and Chun, 2020; Shao et al., 2022). Recently, Shao et al. (2022) reported that lettuce (*Lactuca sativa* L. cv. ‘Zishan’) plants accumulated more biomass under one CL (red and blue). However, with the alternation of temporally overlapped red and blue light biomass accumulation decreased with the increase of alternating light frequency.

### Photosynthetic pigments and photosynthetic light use efficiency

Multiple studies have demonstrated that light intensity, photoperiod and thus DLI influence the synthesis of photosynthetic pigments (Lefsrud et al., 2006; Dou et al., 2018; He et al., 2020a; Kelly et al., 2020; Proietti et al., 2021). Longer photoperiods as well as higher DLI increase plant growth by increasing leaf area and Chl and Car concentrations in kale (Lefsrud et al., 2006) and Chl concentrations in lettuce (Kelly et al., 2020). For sweet potato seedlings, as a PPFD increased from 150 to 250 μmol m^-2^ s^-1^, an increasing trend was observed in total Chl and Car concentrations. However, grown under a PPFD of 350 μmol m^-2^ s^-1^, sweet potato seedlings had lower total Chl concentration compared to other conditions (He et al., 2020a). Both Chl and Car of rocket plants were increased by CL irrespective of the light spectrum (Proietti et al., 2021). Dou et al. (2018) reported that high DLIs resulted in lower total Chl concentration on the basis of leaf FW and higher *Chl a/b* ratios while sweet basil plants adapted to lower DLIs increased Chl synthesis to maximise light capture. In the present study, no significant differences were found in total Chl, total Car concentrations, and the *Chl a/b* ratio among the different light treatments (**Figure 5A, B, C**). Thus, effects of DLI on photosynthetic pigments are species dependent. However, Chl/Car ratio of L3 plants was significantly lower than those of all other groups (**Figure 5D**) due to a higher proportion of Car. It is well known that Car function to dissipate excess energy through the xanthophyll cycle to protect the plants from photodamage (Demmig-Adams and Adams, 1996). L3 plants grown under CL conditions had lower F_v_/F_m_ ratios,<0.8 (**Figure 6A**), indicating of photoinhibition may have occurred in these plants (Rosenqvist and van Kooten, 2003).

Photosynthetic pigments catch light energy which is used for the light reactions to drive photosynthetic electron transfer (Fromme et al., 2003). Plant growth depends on not only light interception but also photosynthetic light use efficiency (Palmer and van Iersel, 2020). Generally, higher Chl concentration increases light absorption which are associated with higher ETR and the effective quantum yield of PSII (ΔF/F_m_’ or ΦPSII) (He and Qin, 2020; Kelly et al., 2020). Both ETR and ΔF/F_m_’ are useful indicators of non-cyclic electron transport for overall photosynthesis (Maxwell and Johnson, 2000; Fu et al., 2012). The greatest ETR and ΦPSII were found in Romaine lettuce grown under a PPFD of 200 μmol m^−2^ s^−1^ compared to the others grown under PPFDs which was either lower or higher than 200 μmol m^−2^ s^−1^ for a same photoperiod of 14 h. These results imply that low and high light intensity and DLI partially account for a reduction of photosynthetic light use efficiency (Fu et al., 2012). Theoretically, only increasing the photoperiod can enhance the net daily photosynthesis after reaching the light saturation point. For example, with increasing PPFD, the ФPSII decreased linearly with DLI increasing from 8.6–20.2 mol m^−2^ d^−1^ (He et al., 2020a). That is why increasing the photoperiod increased plant growth but increasing PPFD did not (Kelly et al., 2020). Elkins and van Iersel (2020) also found that the total electron transport through PS II would increase if the same DLI was provided at a lower PPFD over 24 h. However, in the present study, ETR and ΔF/F_m_’ were the lowest in L3 plants while L2 plants had the highest readings followed by L4 and L1 plants under a PPFD of 1076 m^-2^ s^-1^, (**Figures 6B–D**) despite all plants had similar levels of total Chl and *Chl a/b* ratio (**Figures 5A, C**). This result further supported the fact that L3 plants grown under CL had experienced light stress. It was reported that hyperaccumulation of starch in response to CL may lead to an over-reduction of electron acceptors. This generates high concentrations of reactive oxygen species (ROS), resulting in oxidation of cellular components, decreasing the efficiency of photosynthetic systems (Haque et al., 2015). Under optimal growth conditions, large amount of absorbed light is used to drive photosynthesis, defined as qP or small portion of light dissipated as heat, termed as NPQ. Generally, with the increase of light intensity, qP gradually declines while NPQ gradually increases. In this study, measured under a PPFD of 1076 m^-2^ s^-1^, both qP and NPQ values of L1, L2 and L4 plants were significantly higher than L3 plants grown under CL (**Figures 6D, E**). Higher values of NPQ in L2 and L4 plants compared to that of L1 plants, allowing for excess light energy to be dissipated as heat, conferring photoprotection with higher qP (Müller et al., 2001). Although ETR was lower in L3 plants compared to those of L1, L2 and L4 plants, the total electron transport through PS II would increase for L3 plants under CL (Elkins and van Iersel, 2020). Furthermore, over-reduction of electron acceptors beyond the capacity of NPQ may result from hyperaccumulation of starch in response to L3 plants grown under CL (Haque et al., 2015). Both qP and NPQ values decreased simultaneously in L3 plants demonstrating that utilisation of excess light energy absorbed by leaves of L3 plants through photochemical quenching and heat dissipation decreased in parallel. Thus, more excess energy may be used to generate ROS, which mitigated photoinhibition to certain extent. However, continuous excess light energy and ROS accumulation could destroy photosynthetic apparatus.

### Nitrogen metabolism

Light is a critical factor affecting NO_3_^-^ uptake and N metabolism in plants (Bian et al., 2015). A key step of the NO_3_^-^ assimilation pathway is the reduction of NO_3_^-^ to ${\text{NO}}_{2}^{-}$ , catalysed by NR in the cytosol (Mokhele et al., 2012). In this study, NO_3_^-^ assimilation occurred primarily in the leaves of purslane as they had the highest NRA_max_ under all light conditions compared to the stems (**Figure 8A**). This likely resulted in a lower NO_3_^-^ (**Figure 7A**) and higher TRN concentration (**Figure 7B**) in the leaves than other plant organs. Light intensity has remarkable impacts on ${\text{NO}}_{3}^{-}$ reduction in plants. High light intensity promotes NRA (He et al., 2017b). In this study, all stems had similar NRA_max_ while leaf NRA_max_ of purslane was significantly higher in L2 (320 µmol photon m^-2^ s^-1^) and L4 plants (480 µmol photon m^-2^ s^-1^) than L1 and L3 plants (240 µmol photon m^-2^ s^-1^) (**Figure 8A**). Under high light, carbohydrate accumulation upregulates mRNA transcription of NR, increasing NRA (Lillo, 1994) and upregulating ${\text{NO}}_{3}^{-}$ assimilation. In this study, L2 and L4 plants were exposed to higher PPFDs while L2 and L3 plants were subjected to longer photoperiods (18 and 24h respectively) than L1 and L4 plants (both 12h). In the dark, NO_3_^-^ is generally stored in the vacuoles of the leaves. Only upon light exposure are the stored ${\text{NO}}_{3}^{-}$ remobilised into the cytosol by increased NRA (He et al., 2017b). A decrease in photoperiod could limit the export of NO_3_^-^ into the cytosol of the leaves for the first reaction of NO_3_^-^ assimilation which could decrease leaf TRN concentration of L1 and L4 plants (**Figure 7B**). Despite having a lower leaf NRA_max_ (**Figure 8A**), higher leaf NO_3_^-^ (**Figure 7A**) and TRN concentration (**Figure 7B**) in L3 plant, may result from continuously uptake, transport and assimilation of ${\text{NO}}_{3}^{-}$ under CL condition. The lower leaf NRA_max_ in L3 plants could be a result of NR degradation by photooxidation (Schuster and Mohr 1990; Shimomura et al., 2020) upon CL exposure. However, Proietti et al. (2021) found that CL significantly reduced NO_3_^-^ concentration in rocket leaves, suggesting that CL may increase the NRA and the reduction of NO_3_^-^. Higher levels of carbohydrates accumulated in rocket leaves under CL, may provide a carbon skeleton for nitrogen metabolism, affecting NR at both transcription and translation levels (Bian et al., 2015). While significant differences in leaf NRA_max_ of purslane were found among the different light conditions, the NR_act_ of both leaf and stem did not significantly differ (**Figure 8B**), suggesting that NRA_max_ was not correlated with NR_act._ In the second reaction of NO_3_^-^ assimilation, ${\text{NO}}_{2}^{-}$ is further reduced to ${\text{NH}}_{4}^{+}$ which are utilised to synthesise amino acids and then proteins (Mokhele et al., 2012). Although leaf TRN concentrations were different in plants grown under different light conditions, the leaf TSP concentration was similar (**Figure 7C**). This suggests that while a decrease in photoperiod of L1 and L4 plants can lead to a decrease in TRN concentrations, reduced TRN concentrations are not low enough to induce N deficiency and disrupt protein synthesis in these plantsaab30C2022$.

### Nutritional quality

Special vegetable crops can be cultivated indoors through manipulation of lighting environments to increase the levels of bioactive compounds which can be used as functional foods (Kelly et al., 2022). Purslane used in this study is one of the best candidate crops as it has high content of antioxidants such as proline, ASC and TPC as well as soluble sugars (Uddin et al., 2012; Fernández-Poyatos et al., 2021; He et al., 2021; Pires et al., 2021). Proline which has antioxidant role is greatly accumulated in purslane under abiotic stress, especially osmotic and oxidative stresses (He et al., 2021). In this study, leaf proline concentration was higher in purslane grown under higher DLI such as L2, L3, and L4 compared to those of L1 plants (**Figure 9A**). Proline biosynthesis is linked to photosynthesis (Alvarez et al., 2022). This was further supported by the finding that under the same DLI, proline concentrations of purslane grown under higher PPFDs of L2 and L4 conditions were higher than under the lower PPFD of L3 conditions. Lowered photosynthetic efficiency measured by lower ETR, ΔF/F_m_’ and qP in L3 plants (**Figures 6B–D**) can result in decrease in proline concentrations. It was found that proline concentration is much higher in purslane leaves than in stems. It was also noticed that proline concentrations of both leaves and stems were higher in the plants with higher DLI regardless of light intensity and photoperiods (**Figure 9A**). For both leaf and stem, no significant differences in TSS were observed among the different light conditions (**Figure 9B**). Zhang et al. (2018) reported that higher light intensity could lead to higher sugar concentration in lettuce. Study with lettuce plants, Song et al. (2020) found that soluble sugars increased from 150 μmol m^−2^ s^−1^ to 350 μmol m^−2^ s^−1^ and then decreased at 450 μmol ·m^−2^ s^−1^. Plants grown under CL conditions could have higher concentrations of insoluble sugars which is mainly starch (Demers and Gosselin, 2000). Since starch concentration was not measured in this study, future studies could focus on the effects of different light intensities and durations on purslane starch concentration. In this study, total ASC and TPC were higher in the leaves than the stems under all light treatments (**Figure 9C, D**) suggesting that purslane leaves are of higher nutritional value. It was reported that higher light intensity could lead to higher content of vitamin C (ASC) in lettuce (Zhang et al., 2018). Under the same DLI, Proietti et al. (2021) found that CL significantly enhanced the ASC concentration in the leaves of rocket plants. However, in this study, all purslane plants had similar total ASC concentration in both leaves and stems (**Figure 9C**) regardless of light conditions. Dou et al. (2018) reported that sweet basil (*Ocimum basilicum*) grown under higher DLIs had higher TPC contents than under lower DLIs. In this study, under the same DLI, purslane grown under L3 with CL also had the higher TPC than L2 and L4 plants in both leaves and stems (**Figure 9D**).

Apart from its rich phytochemicals, purslane is also considered a good source of natural dietary minerals such as K, Ca, Mg and Fe (Alvarez et al., 2022). With the exception of K (**Figure 10A**), higher concentrations of Ca, Mg and Fe were found in the leaves as compared to the stems under all light conditions (**Figure 10B–D**). L1 plants grown under the lower DLI with lower intensity and shorter photoperiod had lower leaf Ca, Mg and Fe concentrations compared to other light treatments. In the study with ten different leafy vegetables, Colonna et al. (2016) reported that higher concentrations of K, Ca and Mg were observed under low light intensity than high intensity. However, the K concentration in both leaves and stems of L1plants were similar to those of L2 and L4 plants, which were significantly higher than that of L3 plants. It was reported that the uptake of macro- and micronutrients depends on both species and PPFD. For example, the concentrations of N, P, K, B decreased while the concentrations of Mg, Fe and Mn increased with increasing PPFD in perennial cover crop legumes (Baligar et al., 2020). The differential effects of PPFD and DLI on the mineral nutrient uptake exemplify that other lighting conditions such as light quality could have some effect on the uptake and allocation of specific dietary minerals. For instance, in the study with okra (*Abelmoschus esculentus* L.), red light at 635 nm enhanced the accumulations of Ca and Mg while the highest N content was found in the plants grown under green light and blue light, respectively at 522 nm and 455 nm (Degni et al., 2021). The effects of light intensity, quality, photoperiod and DLI on dietary mineral accumulation and the mechanisms involved are still largely unknown. This merits a further study on the effect of light conditions on dietary minerals in purslane.

## Conclusion

Compared to 10.368 mol m^-2^ day^-1^ (L1 condition), an overall two times increase in DLI, 20.736 mol m^-2^ day^-1^ (L2, L3 and L4 conditions) through either increasing light intensity and/or extending photoperiod, displayed positive effects on the growth characteristics of purslane. Under the same higher DLI (20.736 mol m^-2^ day^-1^), L2 condition is the most suitable lighting strategy for enhancing productivity and quality of purslane. At an early stage of development, light treatments L2 (320 µmol photon m^-2^ s^-1^, 18 h) and L4 (480 µmol photon m^-2^ s^-1^, 12 h) exerted the most positive effects on purslane productivity. At later stages, light treatment L4 was observed to be the most favourable. However, given that high light intensity is often costly and difficult to maintain, the L2 treatment would be more preferable. L3 treatment with continuous light (240 µmol photon m^-2^ s^-1^, 24 h photoperiod), however, resulted in lower shoot and root growth compared to L2 and L4 conditions under the same DLI. A lowered photosynthetic efficiency, as indicated by the lowered ETR, ΔF/F_m_’ and qP values, was also observed in L3 plants. Thus, L3 treatment may not be favourable for the productivity of purslane. Purslane grown under L2 treatment had the highest concentration of proline, ASC and TSS in the leaves and stems, suggesting that purslane grown under L2 treatment is the most nutritious. Furthermore, leaf dietary minerals such as K, Ca, Mg and Fe concentrations were the highest in purslane grown under L2 compared to the other conditions. No clear trends were observed in stem dietary mineral concentrations among purslane plants grown under the different light conditions. Nevertheless, the results of this study provide growers of purslane a better understanding of the optimal light conditions to enhance its productivity and quality.

## Data availability statement

The original contributions presented in the study are publicly available. This data can be found here: https://researchdata.nie.edu.sg/dataverse/he-jie.

## Author contributions

JH initiated and funded the expenses for the project. JH and LQ planned the experiments and carried out some parts of the experiments. SG carried out most measurements, analysed the data and plotted the graphs under supervision of JH and LQ. JH wrote the 1^st^ draft of the manuscript. JH and LQ revised the manuscript. All authors contributed to the article and approved the submitted version.
